# The learning curve for robot-assisted radical cystectomy with total intracorporeal urinary diversion based on radical cystectomy pentafecta

**DOI:** 10.3389/fonc.2022.975444

**Published:** 2022-10-18

**Authors:** Tae Il Noh, Ji Sung Shim, Sung Gu Kang, Jun Cheon, Jong Hyun Pyun, Seok Ho Kang

**Affiliations:** ^1^ Department of Urology, Anam Hospital, Korea University College of Medicine, Seoul, South Korea; ^2^ Department of Urology, Sungkyunkwan University School of Medicine, Seoul, South Korea

**Keywords:** bladder cancer, robot-assisted radical cystectomy (RARC), intracorporeal urinary diversion, pentafecta, learning curve

## Abstract

**Objective:**

To analyze the learning curve for robot- assisted radical cystectomy (RARC) with total intracorporeal urinary diversion (ICUD) in terms of both time efficiency and quality of surgery based on radical cystectomy (RC)-pentafecta.

**Patients and methods:**

We identified 203 consecutive patients who underwent RARC with ICUD of the ileal conduit (IC, 85) and orthotopic neobladder (ONB, 118) performed by a single surgeon between 2011 and 2021. We grouped ten consecutive patients into time-associated blocks according to the operation order. Process efficiency and operation quality were measured based on the surgeon’s console time and attainment/score sum of RC-pentafecta. The overcoming point of the learning curve was defined graphically and statistically.

**Results:**

The mean follow-up period was 44.5 ± 30.7 months. Of the 203 patients, 109 (53.7%) attained the five criteria of RC-pentafecta (ONB vs IC, 50.6% vs. 55.9%, p = 0.35). The attainment rate and sum of the RC-pentafecta score of the third group were not significantly different from those of all patients (40.0% vs. 53.7%, p = 0.369, 4.00 ± 1.05 vs. 4.41 ± 0.75, p = 0.137, respectively), and the proficiency in operation quality was satisfactory in the third group. The console times continually improved and stabilized after the 140^th^ case (IC, 60; ONB, 80), and the attainment rate and sum of the RC-pentafecta were significantly different between before and after the 140^th^ case (p<0.001).

**Conclusion:**

A single surgeon’s learning curve for RARC with ICUD and pelvic lymph node dissection (PLND) showed an acceptable level of proficiency after 30 consecutive cases in terms of the operation quality. However, for an expert surgeon, 140 cases were required to reach a plateau in time efficiency and second leap with the RC-pentafecta. RARC with ICUD and PLND can be performed safely without compromising functional outcomes and complications through sharing and transmission of standardized techniques.

## Introduction

Radical cystectomy with urinary diversion and pelvic lymph node dissection (PLND) is the current gold standard treatment for muscle-invasive bladder cancer (1). Robot-assisted radical cystectomy (RARC) with bilateral PLND has emerged as an approach equivalent to open radical cystectomy (ORC) as a minimally invasive procedure in an effort to reduce morbidity and enhance recovery (2). An international multicenter collaboration study between North American and European high-volume institutions revealed that the incidence of RARC increased from 29% in 2006–2008 to 54% in 2015–2018 (3).

This procedure has been shown to be safe and feasible in a long-term follow-up, with the RAZOR trial providing evidence to support the oncological efficacy of RARC with urinary diversion (UD) and PLND (4, 5). Although early to intermediate perioperative and oncologic outcomes of RARC with UD and PLND appear comparable to those of open surgery, its feasibility during the initial experience remains controversial (6, 7). In particular, RARC with extracorporeal urinary diversion (ECUD) is widely adopted because of the perceived difficulties associated with bowel reconstruction in intracorporeal urinary diversion (ICUD) and concerns about time efficiency. However, a total ICUD approach could be used as a minimally invasive surgery and maximize its advantages following RARC. Minimizing evaporative fluid loss, decreasing estimated blood loss (EBL), reducing the risk of fluid imbalance and pain, and rapid restoration of bowel function are some of the apparent advantages of ICUD (8, 9).

Despite the introduction of minimally invasive techniques and validation of the oncologic equivalency of RARC with total ICUD, adoption of ICUD in clinical practice has been slow owing to its complexity (10). For this reason, several studies have reported on the learning curve of robotic surgery, and there is clearly a learning curve associated with the acquisition of proficiency in robotic surgery (11, 12). However, few studies have evaluated the learning curve using an objective index in RARC with total ICUD and PLND. Thus far, the learning curve has been defined as the operation time and/or “self-declared” time point when the surgeon feels comfortable performing the operation (11). Thus, this procedure needs to be assessed with objective indicators using an accepted standard definition for the learning curve of RARC with total ICUD and PLND.

For the standardized composite method for reporting outcomes of RC that incorporates both perioperative morbidity and oncological adequacy, RC-pentafecta has been proposed (13, 14). The common criteria in the revised proposal were as follows: (1) negative soft tissue surgical margin (STSM); (2) ≥ 16 lymph nodes (LNs) yielded; (3) no major complications over Clavien-Dindo grade 3–5 within 90 days; (4) absence of clinical recurrence within 12 months; and (5) no ureteroenteric strictures requiring intervention. We also validated that RC-pentafecta could be used to standardize the assessment of the surgical quality of RARC from the multicenter Korean Robot-Assisted Radical cystectomy Study Group (KORARC)database (15).

We aimed to show the process to surmount the learning curve to evaluate the quality of operation based on RC-pentafecta and the time efficiency through a single surgeon’s experience. In addition, we describe the standardized techniques and tips for RARC with total ICUD.

## Patients and methods

### Study population

Between January 2011 and January 2021, 203 consecutive patients underwent RARC with ICUD and PLND in a single referral tertiary center performed by a single surgeon for high-grade and/or muscle-invasive urothelial bladder cancer. Utilizing a single surgeon’s robotic cystectomy database, the learning curve was assessed by a standardized quality of operation based on RC-pentafecta and time efficiency. All robot-assisted surgeries were performed using the DaVinci Surgical System Si or Xi (Intuitive Surgical Inc., Sunnyvale, CA, USA). The surgeon in this study started performing the RARC with ECUD as a robotic surgery-naïve surgeon. He had prior experience with open radical cystectomy but did not have any robotic surgery fellowship or mentor surgeons. Prior to performing RARC with total ICUD and PLND, 38 RARC procedures with extracorporeal urinary diversion were performed. The surgeon commenced RARC with extracorporeal orthotopic neobladder (ONB) with Studer type on the 5^th^ case. On the 16^th^ case, he performed extended PLND initially. After gaining experience with 38 cases of RARC with EUCD and around 100 cases of robotic surgeries such as RARP, robot assisted partial nephrectomy and radical nephroureterectomy, the surgeon shifted to performing RARC with ICUD to maximize the advantages of minimally invasive surgery. RARC with total ICUD orthotopic neobladder [ONB, simple U configuration, modified Studer method (16, 17)] was commenced in his 39^th^ case of RARC. Following that, most of procedures were performed with ICUD except for <5 cases who had absolute contraindications for prolonged surgery time with steep Trendelenburg position due to the high intraocular pressure. The surgeon had 15 years of experience after performing more than 1000 RARPs and 300 (annually >40 cases) RARC with total ICUD. All 203 patients who underwent RARC with ICUD for muscle-invasive bladder cancer or high-risk non-muscle-invasive bladder cancer, regardless of pathology, were enrolled to analyze the surgeon’s learning curve in ileal conduit (85) and ONB (118) procedures. We assessed and analyzed the learning curve with time efficiency by grouping 10 patients according to time sequence and the quality of operation based on RC-pentafecta, which is the standard composite method for reporting outcomes of RC that incorporates both perioperative morbidity and oncological adequacy. The related complication rates were analyzed for the total, early, and late periods according to the Clavien-Dindo classification (18).

### Surgical technique

The detailed technique of RARC and PLND have been described previously (19–22).

**Figure 1:**
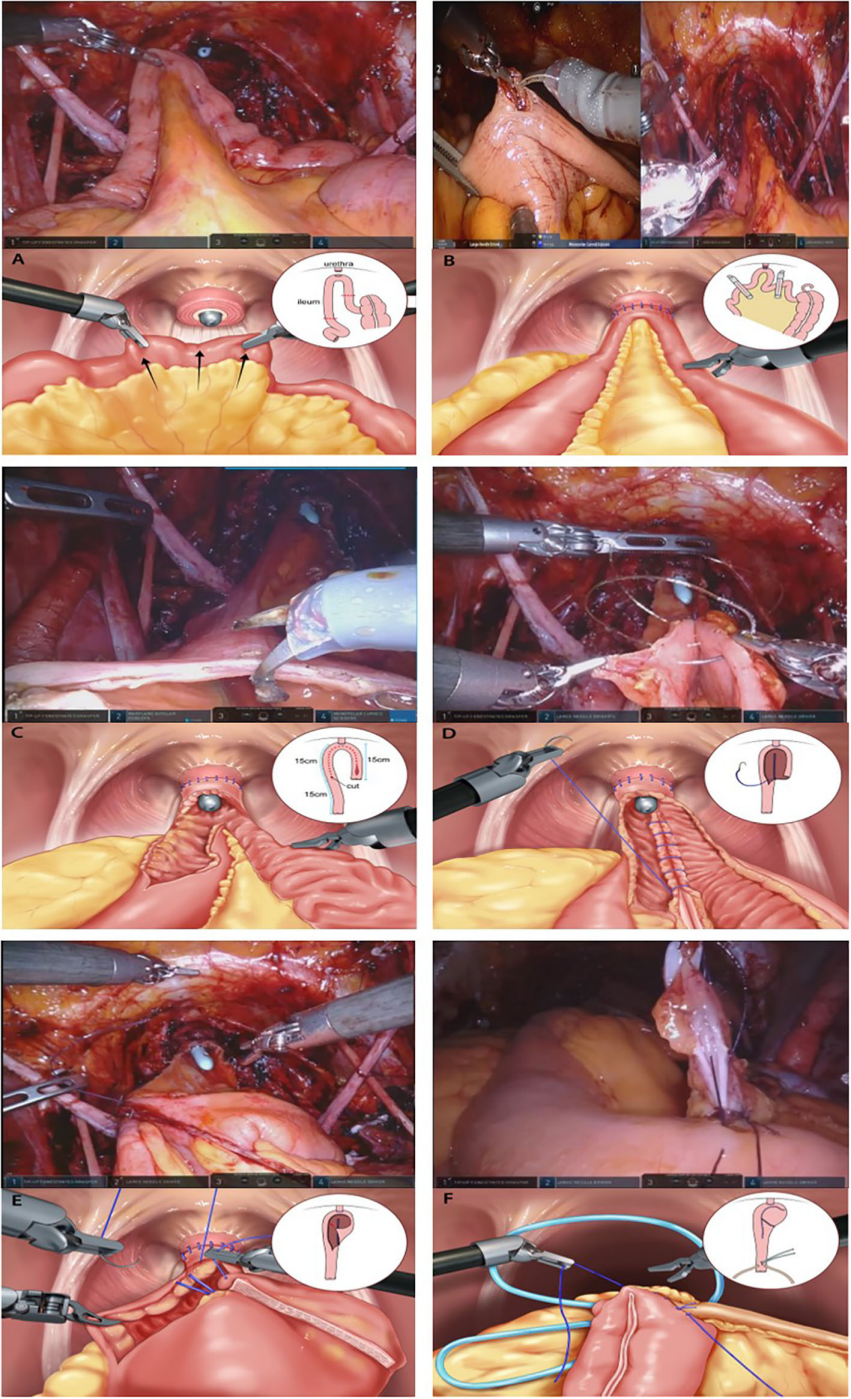
Construction of the modified Studer neobladder **(A)** The 40–45 cm long U-shaped configuration segment is detubularized along the anti-mesenteric border. **(B)** Posterior tagging stay suture with 3-0 polyglycolic acid sutures are applied starting from the far side of the urethra and progress toward the urethral anastomosis site. **(C)** The posterior plate of the neobladder is closed by sewing each medial border with running barbed sutures (Stratafix® 3-0). **(D)** Folding is performed from the right side to the middle of the left side of the anterior detubularized border with 3–0 polyglycolic acid tagging suture. **(E)** The anterior part of the reservoir is also sutured with running barbed sutures (Stratafix® 3-0). **(F)** After closing one side of the ureteroenteric anastomosis, a single-J ureteral stent is inserted into the ureter and the other end of the stent is externalized through the other side of the isoperistaltic afferent limb.

Here, we describe a detailed surgical technique focused on total ICUD (**Figure 1**).

### Detailed surgical technique for orthotopic neobladder

#### Bowel Harvesting

The principle of ileum harvesting is similar to that of traditional orthotopic UD. For the Studer ONB procedure, a 60 cm long segment of the distal ileum was isolated using double-fenestrated graspers, 10–15 cm proximal to the ileocecal valve. The ileum was mobilized to ensure tension-free anastomosis between the urethra and the neck of the neobladder. The modified Studer pouch was measured and created from a 40–45 cm long segment of the distal ileum with a U-shaped configuration for the reservoir, and a 15–20 cm long segment of the proximal ileum was used for the afferent limb.

#### Neobladder-Urethral Anastomosis

Anastomosis was performed between the ileum (future neobladder) and urethra to reduce the use of the robotic arm for traction of the ileum and to maintain stable formation of the neobladder. The proximal 15–20 cm long segment was left for the creation of the afferent limb, and the urethra was anastomosed to the middle of the rest of the segment with absorbable bidirectional barbed suture (Stratafix^®^ 3-0, Ethicon Inc., USA). Posterior reconstruction with a single-arm barbed suture (Stratafix^®^ 3-0), a maneuver similar to the “Rocco stitch,” could help reinforce the anastomosis and reduce the tension between the neobladder and urethra.

#### Detubularization of the Ileum

Except for the bowel segment of the afferent limb, the isolated ileal segment (the measured U-shaped segment [40–45 cm]) was detubularized along the anti-mesenteric border. Insertion of the laparoscopic suction by a tableside assistant and bi-directional traction of the ileum could be helpful in identifying the anti-mesenteric portion easily.

#### Creation of the Neobladder

The 40–45 cm long U-shaped configuration segment was detubularized along the anti-mesenteric border. A posterior tagging stay suture and 3-0 polyglycolic acid sutures were applied starting from the far side of the urethra and progressing toward the urethral anastomosis site. The posterior plate of the neobladder was closed by sewing each medial border using running barbed sutures (Stratafix^®^ 3-0). Folding was performed from the right side to the middle of the left side of the anterior detubularized border with 3-0 polyglycolic acid tagging suture. The anterior part of the reservoir was sutured using running barbed sutures (Stratafix^®^ 3-0).

#### Ureteral Implantation with Ureteral Catheter Insertion

The left ureter was transposed to the right by creating a tunnel under the sigmoid mesentery. Each ureter was spatulated, and a standard bilateral end-to-side anastomosis between the ureter and the isoperistaltic afferent limb was performed using interrupted 4-0 polyglycolic acid sutures on a cutting needle. After one side of the surface was closed with a running suture, a single-J stent (UROSOFT^®^ 6 Fr [2 mm] × 70 cm, Bard, USA) was passed up to the kidney. Subsequently, the other side of the surface was closed. The same procedure was performed on the ureter on the other side.

### Detailed surgical technique for ileal conduit

The principle of ileum harvesting is similar to that of the traditional ileal conduit. A flexible ruler was used to approximate anti-mesenteric ileal borders. A portion of the distal ileum, approximately 10–15 cm long, was chosen, which was 10–15 cm proximal to the ileocecal valve. For the identification of the mesenteric vasculature and blood supply, indocyanine green dye (ICG, quantity calculated based on the patient’s weight) was used. After identifying the vasculature, two sequential firings of endovascular gastrointestinal anastomosis (Endo-GIA) staplers were performed, and the continuity of the open ends of the ileum was established using a single transverse firing of the Endo-GIA stapler, ensuring that anastomosis between both sides of the ileum was secure. Ureteral implantation of the harvested bowel was performed using the same process as that used for the orthotopic neobladder.

### Detailed tips for possible issues

#### Short Bowel Mesenteric Length to Reach the Urethra

**Figure 2:**
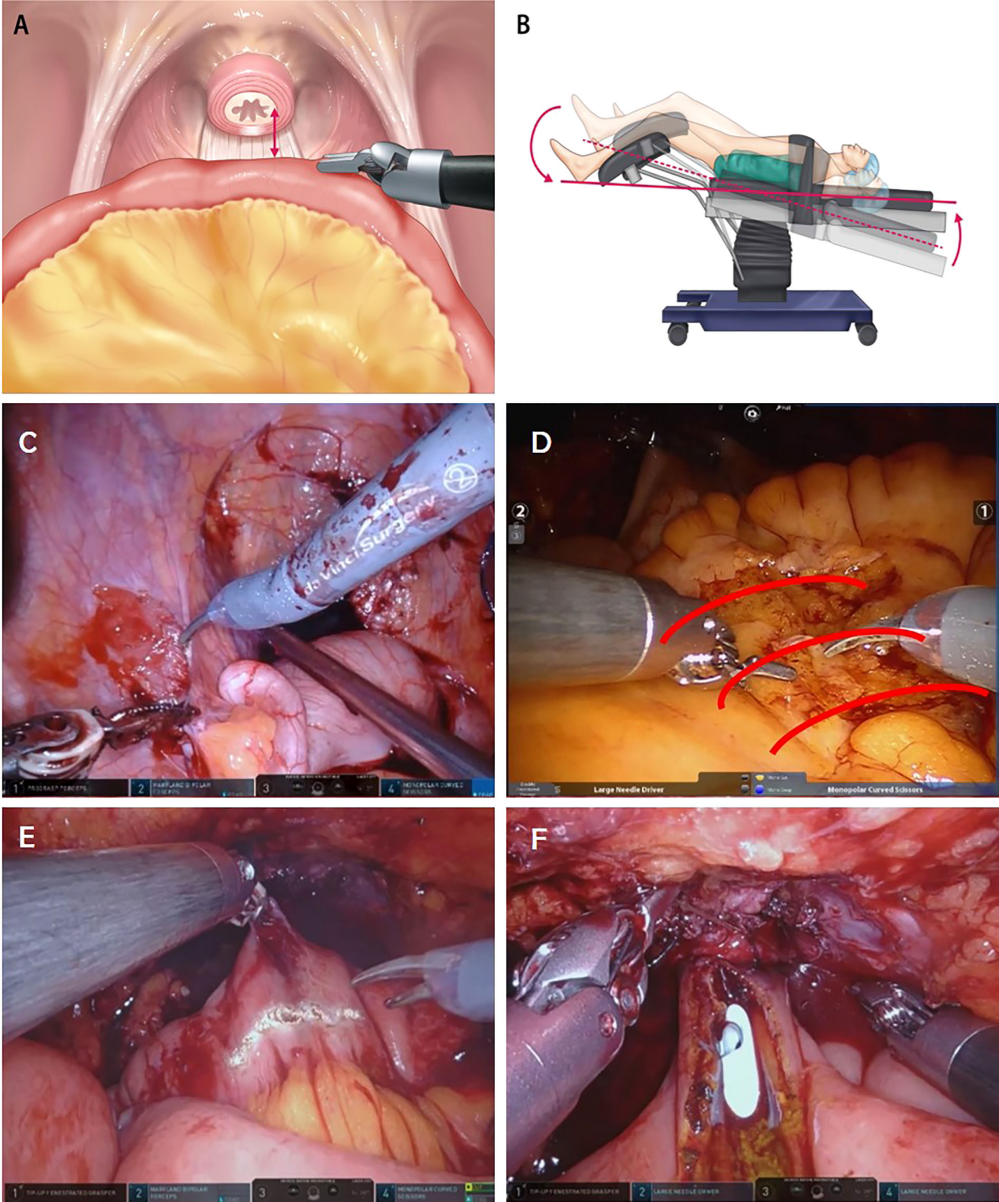
**(A)** The ileum is mobilized to ensure a tension-free anastomosis between the urethra and neck of the neobladder. **(B)** The patient is flattened out of the steep Trendelenburg position from 25° to close to 0°. **(C)** Adhesiolysis of the ileocecal junctional area. **(D)** Creating windows on the intestinal mesenteric border. **(E)** Horizontal incision of ileum (future neobladder) to reach the urethra-neobladder anastomosis site. **(F)** Manual external perineal compression.

The ileum was mobilized to ensure tension-free anastomosis between the urethra and the neck of the neobladder. When the length of the ileal segment to the urethra was sufficient, the Trendelenburg position was maintained. However, when the length of the ileal segment required to create the neobladder (to reach the urethra) is short, several tips and techniques can be used to reduce the tension induced by gravity. Depending on the situation, this tension can be reduced by implementing the first three steps. If the length remains short, the remaining steps are followed or mixed, as follows (**Figure 2**):

1. The robot is undocked, and the patient is flattened out of the steep Trendelenburg position from 25° to close to 0°. After urethroileal anastomosis, the patient is placed in the steep Trendelenburg position again.
2. Perineal compression: In any situation where the short ileal segment is unable to reach the urethra, this simple technique can be easily adopted to augment the length by an additional 1–2 cm.
3. Adhesiolysis of the ileocecal (IC) junctional area: Adhesiolysis of the IC junctional tissues, including the IC folds and line of Toldt, could help draw the mesenteric root to the urethra.
4. Creating windows on the intestinal mesenteric border: Multiple windows on the intestinal mesenteric border from the mesenteric root to the point of urethroileal anastomosis could be helpful in expanding the mesenteric length.
5. Horizontal incision of the ileum (future neobladder) to the urethral anastomosis site: A horizontal incision of the ileum can be used to convert the lumen of the ileal tubule into an additional length. This length is created after ileal detubularization, and urethroileal anastomosis has already been performed; this can also be used to create additional length in advance for urethroileal anastomosis.

These tips can facilitate handling of the bowel while helping to bring the neobladder down to the urethra, decrease traction caused by gravity, and help secure a sufficient length.

#### Insufficient Blood Supply to the Bowel Segment

When poor blood supply to the segment of the ileum was recognized, ICG was injected again to identify this segment. This segment was then resected using the Endo-GIA stapler and sealing devices.

### Definition of robot-assisted radical cystectomy pentafecta

Five criteria were used to define robot-assisted RC-pentafecta to assess the operator learning curve [15]: (1) negative soft tissue surgical margin (STSM); (2) ≥ 16 LNs yielded; (3) no major complication greater than Clavien-Dindo grade 3-5 within 90 days; (4) absence of clinical recurrence within 12 months; and (5) no ureteroenteric strictures requiring intervention. Patients who met all the five criteria were considered to have RC-pentafecta.

### Statistical analysis

We grouped the 10 patients according to the operation order, with a total of 20 groups of 203 patients, with the 20^th^ group consisting of 13 patients. We scored each criterion and analyzed the sum and attainment of the RC-pentafecta points by operation order group. Demographic data, perioperative data for RC-pentafecta, operation time (total operation time/console time), perioperative complications according to the Clavien-Dindo classification (major complications ≥ grade 3, and ureteroenteric strictures requiring intervention), pathologic data (e.g., pathologic stage, LN yield, number of positive nodes, positive surgical margins), and oncologic outcomes (e.g., local and distant recurrence) were collected and analyzed. The available perioperative clinical parameters and outcomes were collected and analyzed using t-test, chi-square test, and Fisher’s exact test. All statistical analyses were performed using IBM SPSS version 24.0 (IBM Corp., Armonk, NY, USA) and R software version 3.6.1 (R Foundation for Statistical Computing, Vienna, Austria). Statistical significance was defined as a two-sided p-value < 0.05.

## Results

The mean follow-up period was 44.5 ± 30.7 months. Of the 203 patients, 171 (85.9%) were men and 32 (15.8%) were women. Urinary diversion was performed in 85 (41.9%) patients with an ileal conduit and in 118 (58.1%) with an ONB. PLNDs were performed in 199 of the 203 (96.0%) patients, standard PLND in 88 (43.3%) patients, and extended PLND in 111 (54.7%) patients. The total mean LN yield (SD) was 24.1 (10.3) and 34.7 (14.8) for standard and extended PLND, respectively. Neoadjuvant chemotherapy was administered to 37 patients (18.2%) and preoperative radiotherapy was performed in 11 patients (5.4%). The basic characteristics and oncological outcomes of our cohort are shown in **Table 1**. The total complication rate classified according to the Clavien-Dindo classification was 62.3%, and the major complication rate for Clavien-Dindo grade 3-5 was 18.7%. Complications are summarized in **Table 2**.

**Table 1:** Baseline characteristics of patients and postoperative outcomes.

	**Total**	**IC**	**ONB**	P-value*
**Number (%)**	203	85 (41.9)	118 (58.1)	
**Age, y,** Mean (SD)	64.74 (10.97)	70.51 (8.25)	60.63 (10.51)	0.043
**Sex, no. (%)**				
Male	171 (84.2)	73 (85.9)	98 (83.1)	0.788
Female	32 (15.8)	12 (14.1)	20 (16.9)	0.358
**BMI,** median (IQR), **kg/m^2^**	24.3 (22.3, 27.2)	25.1 (22.4, 27.2)	24.1 (22.3, 27.2)	0.317
**Clinical and TURBT stage**				
Tis	2 (0.9)	0 (0.0)	2 (1.7)	
Ta	5 (2.5)	3 (3.5)	2 (1.7)	
T1	88 (43.3)	37 (43.5)	51 (43.2)	
T2	95 (46.8)	35 (41.8)	60 (50.8)	
T3	10 (4.9)	8 (9.4)	2 (1.7)	
T4	5 (2.5)	4 (4.7)	1 (0.8)	
**Neoadjuvant, no. (%)**	37 (18.2)	10 (11.8)	27 (22.9)	0.082
**Preoperative radiation, no. (%)**	11 (5.4)	6 (7.1)	5 (4.2)	0.644
**Type of PLND, no. (%)**				
Standard PLND	88 (43.3)	39 (45.9)	49 (41.5)	
Extended PLND	111 (54.7)	42 (49.4)	69 (58.5)	
None	4 (2.0)	4 (4.7)	0 (0.0)	
**Pathologic stage** **after radical cystectomy**				
T0	31 (15.3)	11 (12.9)	21 (17.8)	
Tis	22 (10.8)	5 (5.9)	17 (14.4)	
Ta	7 (3.5)	2 (2.4)	5 (4.2)	
T1	34 (16.7)	15 (17.6)	19 (16.1)	
T2	35 (17.2)	15 (17.6)	20 (16.6)	
T3	55 (27.1)	25 (29.4)	30 (25.4)	
T4	18 (8.7)	12 (14.1)	6 (5.1)	
**Positive surgical margin, no (%)**				
T2	0 (0.0)	0 (0.0)	0 (0.0)	
over T3	4 (1.9)	1 (1.1)	3 (2.5)	
**Yield of LN, no. (%)**				
Standard PLND	24.1 (10.3)	22.3 (10.9)	25.5 (10.5)	0.158
Extended PLND	34.7 (14.8)	32.6 (14.7)	35.9 (15.7)	0.213
**Pathologic nodal stage, no. (%)**				
N0	160 (78.8)	66 (77.6)	94 (79.7)	0.358
N1/N2	37 (18.2)	16 (18.8)	21 (17.8)	0.219
N3	6 (3.0)	3 (3.5)	3 (2.5)	0.114
**Follow-up, mo. mean (SD)**	44.5 (30.7)	52.8 (28.9)	38.5 (20.4)	0.093

P-value * indicates P values for T-test and chi-square test (Fisher’s exact test) between ileal conduit and orthotopic neobladder.

BMI, body mass index; IC, ileal conduit; IQR, interquartile range; ONB, orthotopic neobladder; TURBT, transurethral resection; PLND, pelvic lymph node dissection; LN, lymph node; SD, standard deviation.

**Table 2:** Complications.

	Total (203)	IC (85)	ONB (118)	P value*
Total complication rate (%)	127 (62.6)	52 (61.2)	75 (63.6)	0.381
Clavien gr 1-2	89 (43.8)	32 (37.6)	57(48.3)	
Clavien gr 3-5	38 (18.7)	20 (23.5)	18 (15.3)	
Early complication (first 30 days, % of cases)	73 (36.0)	28 (32.9)	45 (38.1)	0.115
Clavien gr 1-2	60 (29.6)	21 (24.7)	39 (33.0)	
Clavien gr 3-5	13 (6.4)	7 (8.2)	6 (5.1)	
Late complication (30-90 days, % of cases)	54 (26.6)	24 (28.2)	30 (25.4)	0.121
Clavien gr 1-2	29 (14.3)	11 (12.9)	18 (15.3)	
Clavien gr 3-5	25 (12.3)	13 (15.3)	12 (10.1)	
Complications				
Gastrointestinal	53 (26.1)	22 (25.9)	31 (26.3)	0.718
Ileus	50 (24.6)	19 (22.4)	31 (26.3)	
Parastromal herniation	3 (1.5)	3 (3.5)	0 (0.0)	
Infection	48 (23.6)	10 (11.8)	38 (32.2)	0.015
Urinary tract infection	43 (21.2)	9 (10.6)	34 (28.8)	
Sepsis	5 (2.5)	1 (1.2)	4 (3.4)	
Anastomosis related	8 (3.9)	2 (2.4)	6 (5.1)	0.122
Ureteroenteric stricture	8 (3.9)	2 (2.4)	6 (5.1)	
Anastomotic bowel leakage	0 (0.0)	0 (0.0)	0 (0.0)	
Others	18 (8.7)	7 (8.4)	11 (9.3)	0.643
Wound	6 (2.9)	2 (2.4)	4 (3.4)	
Pulmonary	5 (2.4)	2 2.4)	3 (2.5)	
Cardiovascular	4 (1.9)	2 (2.4)	2 (1.7)	
Neurological	3 (1.5)	1 (1.2)	2 (1.7)	

IC, ileal conduit; ONB, orthotopic neobladder.

### Attainment of RC-pantafecta according to the operation order group

**Figure 3:**
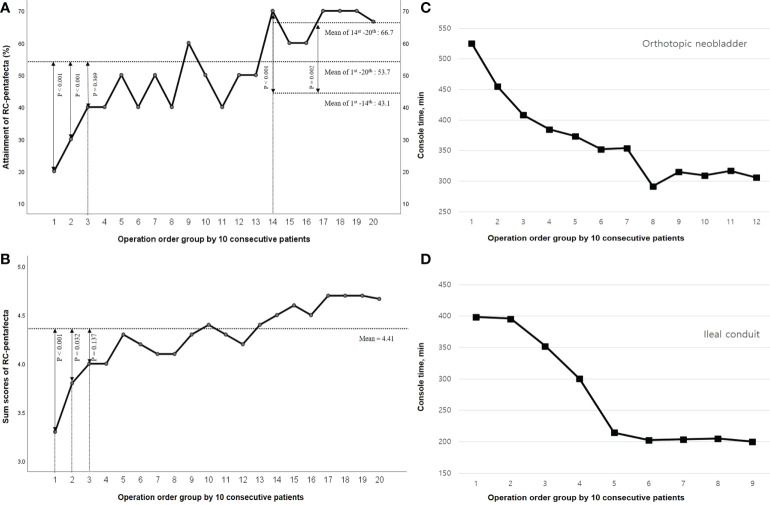
Radical cystectomy (RC)-pentafecta and Console time according to the operation order group. **(A)** Attainment of RC-pentafecta. **(B)** Sum scores of RC-pentafecta. **(C)** Console time of orthotopic neobladder. **(D)** Console time of ileal conduit.

Of the 203 patients, 109 (53.7%) attained five criteria of RC-pentafecta, and the rate of attained RC-pentafecta was not significantly different for ONB and IC (50.6% vs. 55.9%, p = 0.35, respectively). The attainment of all five criteria for RC-pentafecta according to the operation order groups of 10 patients is summarized in **Figure 3A**., which gradually increased according to the operation order group: Group 1, 20%; Group 2, 30%; Group 3, 40%; Group 4, 40%; Group 5, 50%; Group 6, 40%; Group 7, 50%; Group 8, 40%; Group 9, 60%; Group 10, 50%; Group 11, 40%; Group 12, 50%; Group 13, 50%; Group 14, 70%; Group 15, 60%; Group 16, 50%; Group 17, 70%; Group 18, 70%; Group 19, 70%; Group 20, 67% (**Figure 3A**). The mean of RC-pentafecta attainment from the 1^st^ to 140^th^ and 141^st^ to 203^rd^ cases were 43.1% and 66.7%, respectively.

### Sum of the RC-pentafecta score

Negative STSM, ≥ 16 LNs yielded, no major complications more than Clavien-Dindo grade 3-5 within 90 days, absence of clinical recurrence within 12 months, and no ureteroenteric strictures requiring intervention were observed in 95.5%, 83.0%, 79.5%, 85.0%, and 91.5% of patients, respectively. We scored each of the five RC-pentafecta criteria, and the mean sum of each RC-pentafecta score according to the operation order group was 4.41 ± 0.75, gradually increasing with the number of procedures according the operation order group; Group 1, 3.30 ± 0.67; Group 2, 3.80 ± 0.91; Group 3, 4.00 ± 1.05; Group 4, 4.00 ± 1.05; Group 5, 4.30 ± 0.82; Group 6, 4.20 ± 0.79; Group 7, 4.10 ± 1.10; Group 8, 4.10 ± 0.87; Group 9, 4.30 ± 1.06; Group 10, 4.40 ± 0.70; Group 11, 4.30 ± 0.68; Group 12, 4.20 ± 0.98; Group 13, 4.40 ± 0.70; Group 14, 4.50 ± 0.85; Group 15, 4.60 ± 0.52; Group 16, 4.40 ± 0.69; Group 17, 4.70 ± 0.48; Group 18, 4.70 ± 0.52; Group 19, 4.70 ± 0.48; Group 20, 4.67 ± 0.51 (**Figure 3B**).

### Console time

The console times trended down with increasing serial cases. The mean console times (± SD) were 339.07 (± 96.96); IC: 293.79 (± 106.67), ONB: 362.59 (± 82.62). As depicted in **Figures 3C and D**, the implementation time of RARC with ICUD continually improved and stabilized after 60 cases of IC and 80 cases of ONB.

### Learning curve

In terms of objective surgical quality with the RC-pentafecta, the attainment rate and sum of the RC-pentafecta score of the 3^rd^ group were not significantly different from those of the entire patient group (40.0% vs. 53.7%, p = 0.369, 4.00 ± 1.05 vs. 4.41 ± 0.75, p = 0.137, respectively) from the first cases, and the learning curve was overcome in the 3^rd^ (31^th^ to 40^th^) group. In terms of process efficiency, the console times continually improved and stabilized after the 140^th^ case (60 cases of IC and 80 cases of ONB), and the rate of attainment/sum of RC-pentafecta trended increased with increasing serial cases. Noticeably, these attainment of RC-pentafecta scores increased at the 14^th^ group (1^st^ to 13^th^ vs. 14^th^, p < 0.001) and showed a second leap and plateau. After 140 cases, the second learning curve for the expert was overcome when considering both the time efficiency and operational quality.

## Discussion

RARC and PLND have been standardized with comparable oncological efficacy and several advantages (23). ICUD is an attempt to create a minimally invasive urinary diversion following RARC. It has the potential benefit of causing fewer gastrointestinal complications by minimizing bowel manipulation (8). Recently, a three-way comparison study was reported, which included 272, 375, and 301 patients who underwent ORC, ECUD, and ICUD, respectively (24). They analyzed the perioperative outcomes and complications associated with these three surgical approaches. The operative time was shorter for ORC than for ECUD and ICUD (331.5 vs. 421 and 396 min, respectively). The EBL was lower for ICUD than for ECUD and ORC (300 vs. 400 and 700 cc, respectively, p < 0.001). ICUD was also associated with fewer cases of ileus (p = 0.023) and a shorter hospital length of stay (LOS) (p < 0.001). The major complication (Clavien-Dindo grade 3-4) rates of ICUD at postoperative days 30 and 90 were lower than those of ECUD and ORC (16.9% vs. 24.8% and 26.1%, respectively, p =0.015) (24). Another advantage of ICUD is its relatively small incision size (25). Usually, ECUD requires a ≥ 7 cm incision to handle the bowel and create a ureteral anastomosis using an Alexis wound retractor. The incision of the ICUD is smaller than that of the ECUD because only the specimen needs to be extracted in this procedure. Therefore, there are fewer complications related to the wound, such as less pain and improved cosmetic advantages (20).

However, prolonged operation time has been identified as one of the major limitations of RARC with ICUD (26). When ICUD was first introduced, the total operative time was 9–10 h before the learning curve was overcome (27). The International Robotic Cystectomy Consortium (IRCC) suggests that surgeons with adequate experience in performing RARPs are better equipped to overcome the learning curve associated with RARC (28). For reporting of surgical proficiency and learning curve, several factors have been proposed (29–31). The IRCC suggested that an acceptable level of proficiency can be achieved after 30 cases of standard RARC, based on several relatively objective confounding factors: operation time, positive surgical margin rate, LN yield, and estimated blood loss (29). In the EAU Robotic Urology Section Scientific Working group, the learning curve of multicenter surgeons (nine European high-volume centers) who performed RARC with ICUD was reported (30). The authors included 90-d major complications (MC90; Clavien–Dindo grade ≥3), 90-d overall complications (OC90, Clavien–Dindo grades 1–5), operating time (OT), estimated blood loss (EBL), and length of hospital stay (LOS) for suggesting the learning curve, and this needed 137 cases. The other authors proposed a novel “trifecta” combining recurrence-free status, absence of RARC with ICUD-related severe complications, and functional variables (daytime urinary continence) (31). The functional outcome, such as daytime continence, mainly affected surgical proficiency by preservation of urethral length, posterior support, rhabdosphincter and neurovascular bundles of pelvic plexus. The trifecta including daytime incontinence showed an impact on predicting the overall survival (31). Therefore, these variables have been considered displays of surgeons’ experience since they reflect survival, disease stage, and quality of life.

However, these suggested factors and criteria for learning curve are heterogeneous and influenced by multiple factors including the patient’s age, mental status, underlying disease, bleeding tendency with or without antiplatelet agents for the evaluation of LOS and EBL, and an intact and innervated urethral sphincter, urethral length, low-pressure/large-capacity reservoir (>300 ml), absence of bacteriuria, and completeness of voiding for the evaluation of functional outcomes (32). We also attempted to assess the learning curve using these traditional methods and suggestive factors; however, this was restricted by heterogeneity among the cases (22). Therefore, to date, no accepted standard definition with an objective indicator for the “learning curve” exists, which has been defined as a “self-declared” time point when the surgeon feels comfortable performing the operation (11).

Recently, a standardized composite method for reporting the outcomes of RC that incorporates both perioperative morbidity and oncological adequacy, the RC-pentafecta criteria, has been proposed and validated (13–15). We evaluated the learning curve of RARC with ICUD and PLND approaches with objective indicators to assess the quality of operation with RC-pentafecta as well as the analysis of time efficiency.

During the course of consecutive 203 RARC with ICUD by a single surgeon’s series, the authors demonstrated that RARC with urinary diversion, especially the total intracorporeal approach, could be performed safely without compromising perioperative outcomes and complications as well as oncologic results, while there were meaningful improvements in time efficiency and quality of operation based on RC-pentafecta. In this study, approximately 30 cases were needed to reach acceptable surgical quality during the initial experience period, which is in line with the IRCC suggestion for 30 cases (31). However, the console time gradually improved and stabilized after the 140^th^ case (60 IC and 80 ONB). Furthermore, the attainment and sum score of the RC-pentafecta showed a second leap and plateau after the 14^th^ group.

We defined 140 cases as the second learning curve for an expert who overcame this initial learning curve. Clinically, many urologists mentioned that a particularly difficult part of RARC, UD, and PLND is bowel manipulation, which is related to concerns about time efficiency compared to open surgery (6). RARC with total ICUD is time-consuming owing to its high complexity with bowel manipulation and difficult situations due to the short length of the bowel mesentery and recognition of insufficient blood perfusion at the harvested bowel segment. For acquiring a proficiency level in ICUD, especially ONB, the procedures that surgeons should be most familiarized with are bowel harvest, urethral anastomosis, neobladder formation, and ureter implantation in intracorporeal status. Therefore, to solve unexpected issues during ICUD with bowel manipulation even after the initial learning curve (30 cases), it is necessary to set a suitable surgical configuration and solutions for each trouble situation through constant trial and error. The second learning curve should be overcome through these established processes for each situation during the operation. Several tips for troubleshooting related to the short length of the bowel and insufficient blood perfusion at the harvested bowel segment could be applied to shorten the period of the second learning curve.

This study has potential limitations, including the lack of a reference to overcome the learning curve, the significant heterogeneity in case complexity, and other potentially confounding covariates. However, the attainment of the RC-pentafecta rate in a previously published study was 53.3% in 270 patients (14). In another study, the attainment of the RC-pentafecta rate of RC-pentafecta was 39.4% in 104 patients, which was performed by six surgeons (33), whereas the result of our group was 28.5% in 730 patients by 21 surgeons (15). The lower the number of RARCs performed per surgeon, the lower the attainment of the RC-pentafecta. Therefore, referring to the results (53.3%) of a relatively large-scale study with 270 RARC cases reported by Cacciamani et al. (14), the RC-pentafecta attainment rate in the current study (53.7%) may be a reasonable reference for the learning curve. In addition, this study was an evaluation based on the experience of a single surgeon, and the results may not be generalizable to other surgeons performing RARC. However, the value of this learning curve shows the process of overcoming the learning curve and describes how proficiency can be achieved through completing serial cases. As discussed above, RARC with ICUD is a complicated and time-consuming technique with a significant learning curve that needs to be overcome. Based on these results, leading hospitals have been involved globally in presenting the evolution of this technique to overcome the learning curve and obstacles associated with this procedure. With respect to the RARC with ICUD approach, it could be used worldwide by surgeons to overcome the learning curve by standardizing and simplifying surgical steps using well-established techniques and suitable tips.

Based on our experience, the learning curve analysis of a single surgeon showed that an acceptable level of proficiency could be achieved after 30 cases. A second leap in terms of process efficiency and operation quality was observed after 140 cases of RARC with ICUD. This report helps to define the learning curve for RARC of robotic surgeries and demonstrates an acceptable level of proficiency for RARC with the total ICUD approach.

## Data availability statement

The original contributions presented in the study are included in the article/supplementary material. Further inquiries can be directed to the corresponding authors.

## Ethics statement

This study was conducted in accordance with the Declaration of Helsinki and current ethical guidelines. The study was reviewed and approved by the ethics committee and institutional review board of the Korea University Anam Hospital (IRB No. 2019AN0360). Written informed consent was obtained from all study participants prior to enrolment.

## Author contributions

TN: protocol/project development, data collection, data analysis, manuscript writing. JS: protocol/project development. SuK: protocol/project development. JC: protocol/project development. JP: protocol/project development, data collection, supervision. SeK: protocol/project development, data collection, supervision.

## Acknowledgments

This study was supported by grants from the Korea University College of Medicine.

## Conflict of interest

The authors declare that the research was conducted in the absence of any commercial or financial relationships that could be construed as a potential conflict of interest.

## Publisher’s note

All claims expressed in this article are solely those of the authors and do not necessarily represent those of their affiliated organizations, or those of the publisher, the editors and the reviewers. Any product that may be evaluated in this article, or claim that may be made by its manufacturer, is not guaranteed or endorsed by the publisher.
