# Corrigendum: The learning curve for robot-assisted radical cystectomy with total intracorporeal urinary diversion based on radical cystectomy pentafecta

**DOI:** 10.3389/fonc.2023.1185908

**Published:** 2023-03-23

**Authors:** Tae Il Noh, Ji Sung Shim, Sung Gu Kang, Jun Cheon, Jong Hyun Pyun, Seok Ho Kang

**Affiliations:** ^1^ Department of Urology, Anam Hospital, Korea University College of Medicine, Seoul, South Korea; ^2^ Department of Urology, Kangbuk Samsung Hospital, Sungkyunkwan University School of Medicine, Seoul, South Korea

**Keywords:** bladder cancer, robot-assisted radical cystectomy (RARC), intracorporeal urinary diversion, pentafecta, learning curve

## Incorrect Affiliation

In the published article, there was an error in affiliation 2. Instead of “Department of Urology, Sungkyunkwan University School of Medicine, Seoul, South Korea”, it should be “Department of Urology, Kangbuk Samsung Hospital, Sungkyunkwan University School of Medicine, Seoul, South Korea.”

The authors apologize for this error and state that this does not change the scientific conclusions of the article in any way. The original article has been updated.

